# Clinical utility of the over-ground bodyweight-supporting walking system Andago in children and youths with gait impairments

**DOI:** 10.1186/s12984-021-00827-1

**Published:** 2021-02-08

**Authors:** Hubertus J. A. van Hedel, Irene Rosselli, Sandra Baumgartner-Ricklin

**Affiliations:** 1 Research Department, Swiss Children’s Rehab, University Children’s Hospital Zurich, Mühlebergstrasse 104, 8910 Affoltern am Albis, Switzerland; 2 Children’s Research Centre, University Children’s Hospital Zurich, Steinwiesstrasse 75, 8032 Zurich, Switzerland; 3 Department of Health Sciences and Technology (D-HEST), ETH Zurich, Zurich, Switzerland

**Keywords:** Cerebral palsy, Rehabilitation technology, Pediatric neurorehabilitation, Stride-to-stride variability, Bodyweight unloading, Over-ground bodyweight support, Practicability, Feasibility

## Abstract

**Background:**

The Andago is a rehabilitation robot that allows training walking over-ground while providing bodyweight unloading (BWU). We investigated the practicability, acceptability, and appropriateness of the device in children with gait impairments undergoing neurorehabilitation. Concerning appropriateness, we investigated whether (i) stride-to-stride variability of the stride time and inter-joint coordination was higher when walking over-ground in Andago versus treadmill walking, and (ii) activation of antigravity leg muscles decreased with higher levels of BWU.

**Methods:**

Eighteen children and adolescents with gait impairments participated in three sessions. Practicability was assessed by determining the time needed to get a patient in and out of Andago, the accuracy of the BWU system, and other aspects. Acceptability was assessed by patients responding to questions, while six therapists filled out the System Usability Scale. To determine appropriateness, the participants were equipped with surface electromyography (sEMG) electrodes, electrogoniometers and accelerometers. Various parameters were compared between walking over-ground and on a treadmill, and between walking with three different levels of BWU (median: 20%, 35% and 50% of the bodyweight) over-ground.

**Results:**

Practicability: the average time needed to get in and out of Andago amounted to 60 s and 16 s, respectively. The BWU system seemed accurate, especially at higher levels. We experienced no technical difficulties and Andago prevented 12 falls. However, participants had difficulties walking through a door without bumping into it. Acceptability: after the second session, nine participants felt safer walking in Andago compared to normal walking, 15 preferred walking in Andago compared to treadmill walking, and all wanted to train again with Andago. Therapists rated the usability of the Andago as excellent. Appropriateness: stride-to-stride variability of stride duration and inter-joint coordination was higher in Andago compared to treadmill walking. sEMG activity was not largely influenced by the levels of BWU investigated in this study, except for a reduced M. Gluteus Medius activity at the highest level of BWU tested.

**Conclusions:**

The Andago is a practical and well-accepted device to train walking over-ground with BWU in children and adolescents with gait impairments safely. The system allows individual stride-to-stride variability of temporospatial gait parameters without affecting antigravity muscle activity strongly.

*Trial registration:* ClinicalTrials.gov Identifier: NCT03787199.

## Background

Children and adolescents with neuro-orthopedic disorders can have multiple sensorimotor impairments, limiting their ability to walk. Independent mobility, though, is important for the developing child as it allows exploring its surroundings and enhance socioemotional expression and interaction with the caregivers and other family members [1]. Autonomous mobility is also relevant for enabling independence in many daily life activities and participation.

Consequently, improving gait is an important rehabilitation goal for the patient and his/her social environment. For this reason, rehabilitative interventions often focus on (re-)learning to walk. Depending on the severity of the patient’s impairments, physical support needs to be adjusted, for example, by having one or two physiotherapists keeping the patient upright while assisting leg movements and preventing falls, perhaps in combination with the use of walking-aids like parallel bars or walkers.

For several years, rehabilitation specialists can complement conventional therapies with rehabilitation technologies. For patients with severe impairments, robot-assisted gait training, or manually assisted gait training on a treadmill can be applied. Here, patients receive physical support to assist the leg movements by a device, or by therapists, respectively. The bodyweight support system ensures safety (against stumbling and falling) and facilitates leg movements due to reduced loading of the legs. If the patient improves in leg joint control, but still has difficulties in full weight-bearing, the mechanical or manual assistance could be stopped, and the patient could train further on a treadmill with bodyweight unloading (BWU). Recently, rehabilitation devices that allow walking over-ground with bodyweight support have been developed (for some systems, see https://www.neurorehabdirectory.com/product-category/body-weight-support/). One group of these over-ground BWU systems is designed to be used in a single room as the systems are mounted to the ceiling. Examples are the ZeroG (Aretech, VA USA) [2], SafeGait 360 (Gorbel Medical, NY USA), Vector Gait and Safety System (Bioness, CA USA), or the Ceiling-Mounted Track System (Solo Step, SD USA). One disadvantage of these systems is that they restrict walking spatially, as the patients have to walk more or less following the track. Other ceiling-mounted systems like the RYSEN [3] (Motek, Amsterdam, Netherlands) or FLOAT [4] (Reha-Stim Medtec AG, Schlieren, Switzerland) allow moving around more freely but are still limited to a room. The second group of over-ground BWU systems, namely mobile systems, provide support without being restricted to a room. Examples are the CP Walker [5, 6], the KineAssist [7], the Bungee Mobility Trainer (NeuroGymTech, Ottawa, Canada), or the Andago V2.0 (Hocoma AG, Volketswil, Switzerland).

We received a prototype of the Andago in 2016, later followed by the CE certified commercial version (Andago V2.0). After our robotic team had gathered clinical experience in applying the device, we decided to investigate the clinical utility of the Andago V2.0 in a sample of patients who aimed to improve their ability to walk in our center.

In line with Smart [8], we understand clinical utility as a multi-dimensional model that outlines four factors in practitioners’ and patients’ judgments: practicability, acceptability, appropriateness, and accessibility. Practicability refers to whether the system works well. Acceptability covers ethical, legal, social, or psychological concerns that may affect practice or treatment. Appropriateness includes evidence of effectiveness and relevance for clinical decision-making. Finally, accessibility, i.e., costs and cost-effectiveness or availability and supply of the technology, is an important component of clinical utility but was not investigated in this study.

The overall aim of this study was to investigate several practicability and acceptability aspects of the Andago and to answer two specific research questions covering aspects of appropriateness. Based on the observations made in the therapies and on the literature, we formulated these research questions as follows: the first question was based on our clinical observation that patients who train with BWU on a treadmill walk differently in this condition than when they walk with their regular walking aids over-ground. On a treadmill, variability between steps seems small. The gait pattern appears rhythmical and repetitive and hard to vary and to adapt. When patients leave the treadmill and walk over-ground again with their regular walking aids, the quality of the gait pattern seems to deteriorate as the variability between steps increases, and the pattern becomes less rhythmical and repetitive. This has also been described for healthy young adults [9] and adult patients with hemiparetic stroke [10]. As these differences might be explained by differences in BWU, trunk stability, or the externally triggered movements by the treadmill, we formulated the first research question as follows: (1) is stride-to-stride variability of the stride time and lower limb inter-joint coordination higher when walking over-ground in Andago compared to treadmill walking at similar levels of BWU and velocity?

Furthermore, we know from previous studies that BWU can affect the level of muscle activity [11, 12]. To avoid that training with too high levels of BWU might reduce the training effect, we formulated our second research question as follows: (2) Do levels of antigravity leg muscle activity decrease with higher levels of BWU? Results from this study should help therapists in their clinical decision making whether the Andago might be a therapeutic option for a specific patient or not.

## Methods

### Participants

We included in- and outpatients from the Swiss Children’s Rehab, University Children’s Hospital Zurich, Affoltern am Albis, Switzerland. In line with recommendations for clinical utility studies, which propose 8 to 25 participants [13], we aimed to include 15 to 20 participants. Inclusion criteria were neuro-orthopedic disorders, taller than 125 cm, able to understand simple instructions, able to walk ten meters with or without walking aid, younger than 18 years (a minimum age was not defined), and informed consent. Exclusion criteria were unconsolidated fractures or bone fragility of the lower extremities, skin lesions in the harness’ area which could not be protected, unstable hip, knee, and/or ankle joints, reduced head control or inability to maintain an upright position, inability to communicate discomfort or pain, surgery of the lower extremities in the last three months, recently implanted baclofen pump, implanted pacemakers, passive knee extension deficit > 30°, and self-selected walking speed in Andago > 3.2 km/h. According to the manufacturer, participants should not exceed a bodyweight of 135 kg and a body height of 2 m [14].

Patients were characterized by gender, age, height and –weight, diagnosis and severity, more affected side, and use of orthoses and other walking aids. Further, in line with the International Classification of Functioning, Disability and Health—Children and Youth (ICF-CY) version, we assessed on the body function level, leg muscle strength with the Manual Muscle Test (MMT), and selectivity using the Selective Control Assessment of the Lower Extremity (SCALE).

The MMT is a clinical standard to assess the strength of muscles (or muscle groups) against gravity and manual resistance [15]. The score ranges between zero (‘no contraction palpable’) and five (‘motion in the full range of motion against gravity and maximal resistance’). In this study, bilateral hip flexors and extensors as well as hip internal and external rotators, knee flexors and extensors, and ankle plantar- and dorsal-flexors were tested. We presented a sum score describing overall leg muscle strength, where the maximal score could vary between 0 and 80.

The SCALE is a valid and reliable clinical assessment tool used to evaluate selective voluntary motor control in children with cerebral palsy (CP) [16]. It tests the hip and knee flexion–extension, ankle dorsiflexion-plantarflexion, subtalar inversion-eversion, and toe flexion–extension. The score varies between zero (‘unable’) and two (‘normal’) and depends on the active range of motion and the occurrence of involuntary movements in other joints. The sum score varies between 0 and 20.

On the ICF-CY activity capacity level, patients performed the 10-m walk test (10MWT) at self-selected speed. The 10MWT is a valid and reliable tool to assess the walking speed over a short distance [17, 18]. Participants received standardized instructions to walk at their self-selected speed (with their normal walking aid and orthoses if used in daily life) along a 14-m walkway. The 10 m in the middle were stopped with a stopwatch (i.e., steady-state, so without acceleration and deceleration). Participants performed the 10MWT twice, and the average time of the two trials was reported.

On the ICF-CY activity performance level, we scored the patient’s walking performance in daily life with the Functional Mobility Scale (FMS) and the Gillette Functional Assessment Questionnaire Walking Scale (GFAQ). These are valid and reliable assessments in young patients with neuro-orthopedic disorders [19, 20]. The FMS scores the habitual mobility strategies considering the assistive devices the child uses for distances of 5, 50, and 500 m. The performance for each distance is rated on a 6-point ordinal level varying from 1 (‘uses wheelchair’) to 6 (’independent on all surfaces’). The GFAQ describes with a value between 1 (‘cannot take any steps at all’) and 10 (‘walks, runs, and climbs on level and uneven terrain and does stairs without difficulty or assistance. Is typically able to keep up with peers’) the daily functional mobility of the child.

Finally, we assessed the mobility and cognition subscales of the Functional Independence Measure for Children (WeeFIM) [21]. The WeeFIM contains 18 items covering the subscales self-care, mobility, and cognition, which can be assessed by observing a child’s daily life performance and score this according to criterion standards. For this study, we focused on the mobility subscale with 5 items (3 locomotion and 2 transfer items) and the cognition subscale with 5 items (2 communication and 3 social and cognitive items). Each item is scored from 1 (total assistance) to 7 points (complete independence). The WeeFIM is routinely assessed by trained and certified nurses in our center.

### Andago robotic device

**Fig. 1:**
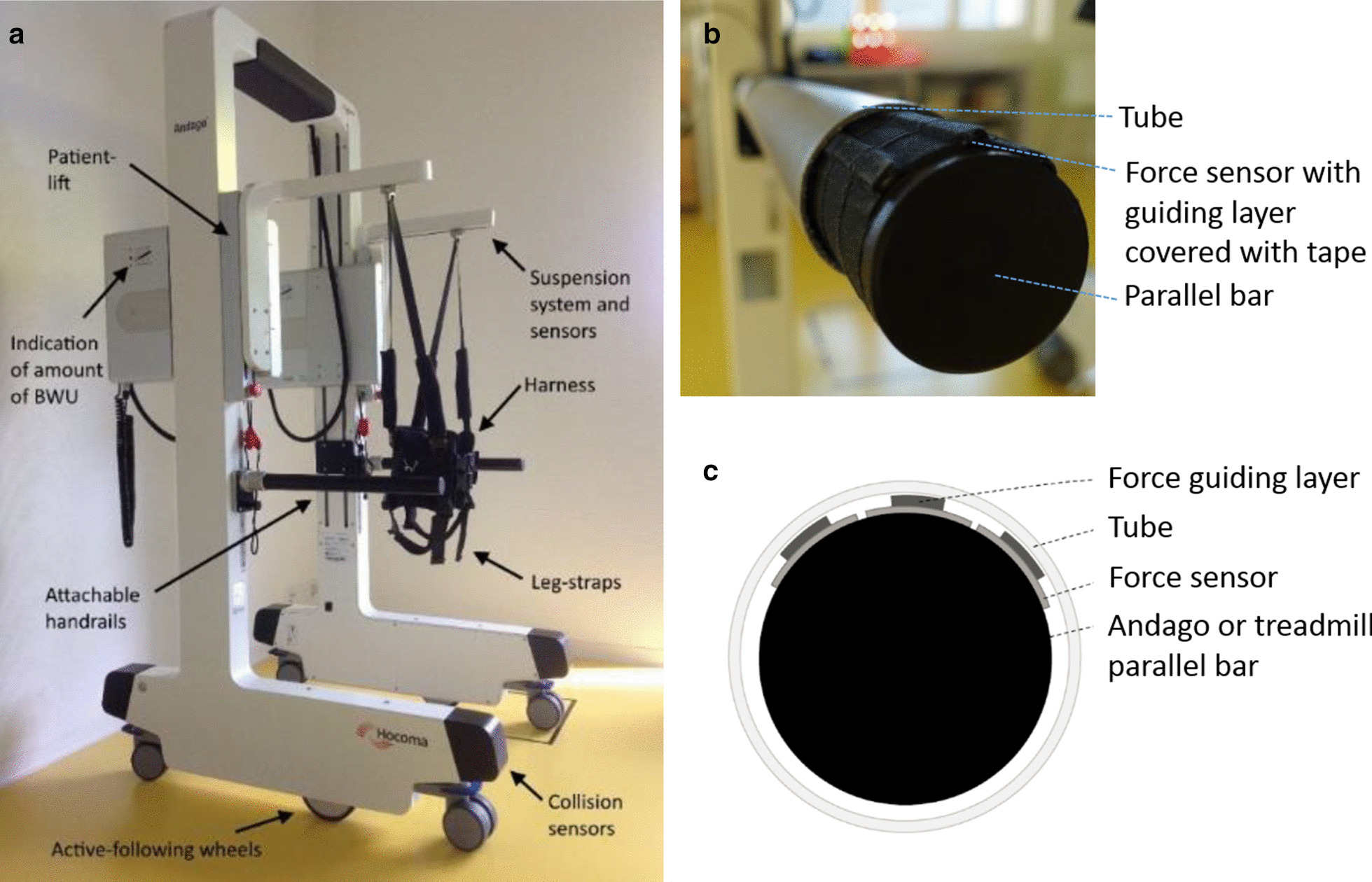
Andago and custom-made pressure detection system. **a** Andago with its various components. **b** Photo, and **c** schematic drawing of handrail equipped with custom-made pressure force detection system. The middle band of each sensor was covered with a stripe (‘force guiding layer’) to increase the thickness of the sensor in the recording area. Sensors and stripes were covered with tape (to improve robustness), and a tube was inserted around the bar to decouple support and grasping forces. *BWU* bodyweight unloading

Andago (V2.0) is a patient-following robotic BWU system for over-ground gait rehabilitation with few spatial limitations (Fig. 1a). Patients are secured with a harness connected to a dynamic BWU system ensuring the safe practice of walking and balance exercises. The Class IIa device weighs 185 kg and measures 107 cm (front-back) by 195 cm (height) by 85 cm (width; inner width is 67 cm), which makes it slender enough to pass through standard doors. Between the harness and the device, sensors are mounted that register the force and direction in which the patient is walking (Fig. 1a). The sensors, in combination with the motorized wheels, allow the robotic system to follow the patient. The dynamic bodyweight support system can be set from 0 to 55 kg by the therapist while the patient is standing upright. It remains constant during the entire gait cycle unless the therapist decides to increase or decrease the support by a few kilograms, which can be done without stopping. However, a stop is needed to readjust the dynamic unloading range. The system protects against and registers potential falls. The robot has two detachable handrails, and sensors integrated in the front of the device to detect collisions, and a patient lift for the transfer from and to the wheelchair (Fig. 1a).

Andago adapts its speed to the patient who can accelerate, decelerate, stop, turn, and even walk backward at any time during the therapy. The system is limited to a maximal speed of 3.2 km/h. It has three different control modes: (i) ‘patient-following mode’: the device follows the movements of the patient in any direction, (ii) ‘straight-line mode’: walking is limited to a straight line (backward or forward), and (iii) ‘manual mode’: the therapist can steer the device with a remote controller. In this modus, the sensors are deactivated, and the system does not follow the patient’s movements [22].

### Study protocol

**Fig. 2:**
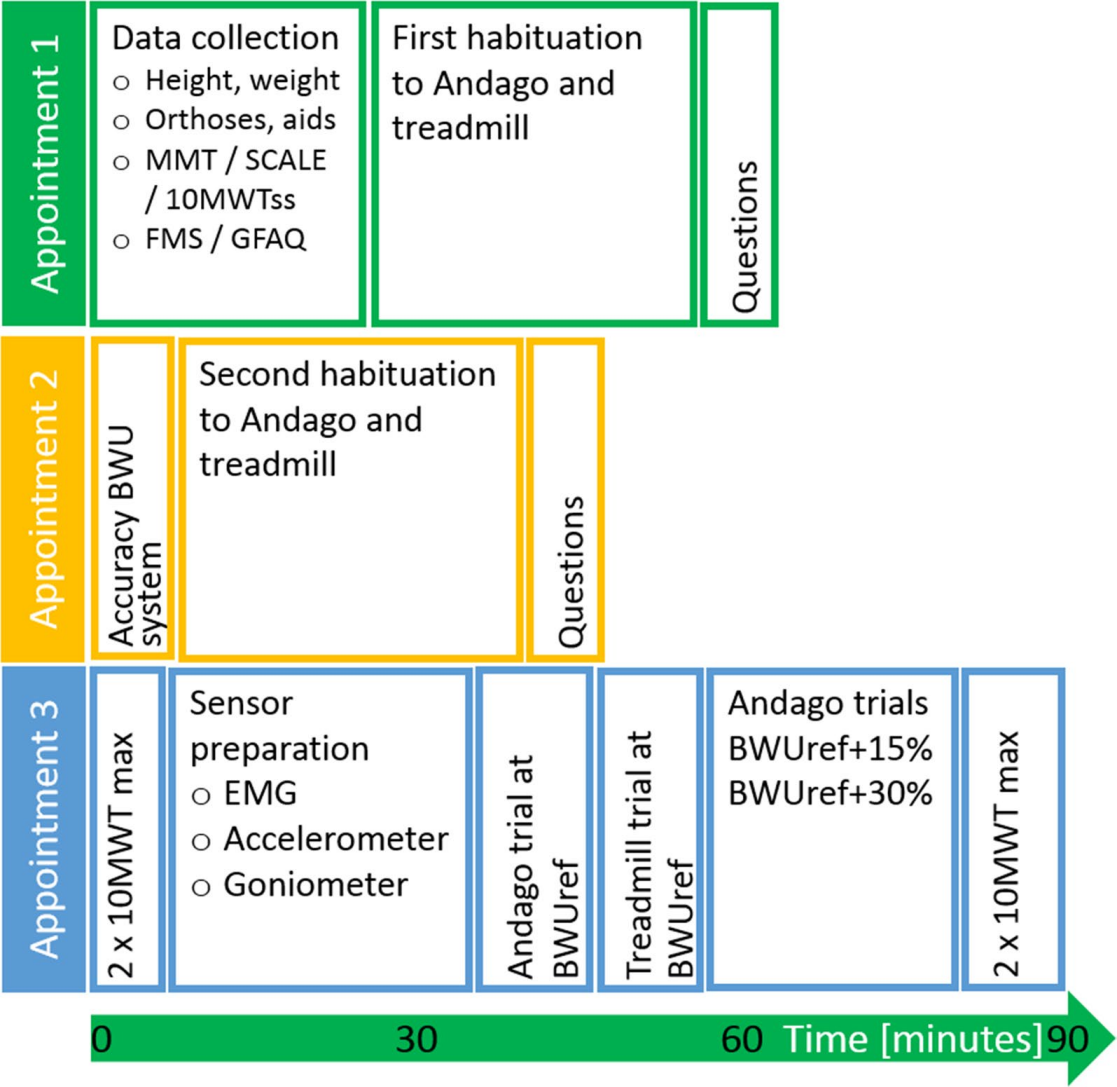
Study procedures. Schematic drawing showing the study procedures. At appointment 1, patient characteristics and functional measures were assessed. Participants were able to practice walking with Andago and on the treadmill. Finally, some questions were asked. At appointment 2, we evaluated the accuracy of the bodyweight unloading system, allowed practicing, and again asked participants about their acceptability of Andago. At appointment 3, participants performed first the 10 MWT at maximal speed twice. Then, EMG electrodes, accelerometers, and goniometers were mounted, after which the measurements in Andago and on the treadmill were performed at the reference bodyweight unloading level (BWUref). This was followed by walking in Andago with the two other BWU levels. Finally, the 10MWT at maximal speed was repeated twice again. *MMT* manual muscle testing, *SCALE* selective control assessment of the lower extremity, *10MWTss* 10-m walk test at self-selected speed, *10MWT max* 10-m walk test at maximal speed, *BWU* bodyweight unloading, *FMS* functional mobility scale, *GFAQ* Gillette functional assessment questionnaire walking scale

Children and adolescents with various diagnoses and gait impairments participated in three sessions (lasting 60, 45, and 90 min, respectively) in our center (Fig. 2). The first two appointments were provided, amongst others, to become familiarized with walking in the Andago and on the treadmill.

In session 1, we assessed the patients’ characteristics and various functional tests (MMT, SCALE, etc.) during the first 25 min. Patients then walked in the Andago (20 min) and on the treadmill (10 min). When training in the Andago, participants practiced the following tasks: 2 × 30 m walking in a straight line (once in the straight line and once in the patient-following mode), 2 × 20 m backward walking in the straight-line mode, 1 × 30 m walking in the straight-line mode forward without using the bars of the Andago, and finally, passing 5 times through a door (width 110 cm) in the patient-following mode. On the treadmill, children walked forward (60 steps) while holding the parallel bars with both hands, followed by 60 steps with one hand on a parallel bar and finally walking forward without holding the parallel bars. The session was closed by a short questionnaire where the children responded to acceptability questions about the system (5 min). In session 2, we investigated the accuracy of the Andago unloading system (10 min), again followed by 30 min of training in the Andago and on the treadmill with the same protocol as in session 1. The acceptability questions of session 1 were repeated at the end of this session (5 min). In session 3, participants were equipped with surface electromyography (sEMG) electrodes, accelerometers, and goniometers (25 min), after which they walked 15 min in Andago and 10 min on the treadmill. This was followed by 20 min of walking over-ground in the Andago at various BWU conditions. At the onset and end of this session, participants performed the 10MWT at their maximum speed twice to control for factors such as fatigue.

Furthermore, all therapists performing therapeutic training sessions with the Andago filled out the System Usability Scale (SUS) questionnaire, which reports on the practicability of the system from the therapists’ perspective [23].

### Practicability

We investigated the following practicability issues:

- Time needed to get the patient in and out of Andago. During sessions 1 to 3, we stopped the time needed to position each participant in the Andago harness, and, at the end of the session, we recorded the time to get the patient out of the device. We calculated the average time for each participant over the three sessions.
- Accuracy of the bodyweight support system. As the bodyweight of some of the children is relatively low, and the Andago was not primarily developed for children, we considered it important to determine the accuracy of the weight support system. We tested three conditions, 25%, 50%, and 75% of BWU. Participants were standing in the Andago in the dynamic BWU range on a digital weighing scale. The Andago weight support was set at 25%, 50%, or 75% (randomized order) of their bodyweight as accurately as possible. We compared the weight based on the Andago settings with the weight displayed on the digital scale [kg]. Besides the absolute differences, we calculated also relative deviations [(Weighing scale—Andago BWU setting)/Andago BWU setting × 100%].
- During the first two sessions, we recorded the number of device deficiencies, the number of prevented falls, the number of safety stops (caused by too rapid movements of the participant), and the number of bumps when passing 5 times through the door in patient-following mode.

### Acceptability

We quantified the young patients’ acceptability of Andago by asking (1) whether they felt safer walking in Andago or walking normally, (2) whether they would prefer to train walking in Andago or on the treadmill, and (3) whether they would like to walk again in Andago. Furthermore, we asked how cool it was to train in Andago (Likert scale 0, i.e., not cool at all, to 10, i.e., very cool). We asked these questions at the end of sessions 1 and 2 (Fig. 2).

Therapists who applied the Andago during regular clinical therapies in our center filled out the SUS. It comprises ten questions that are scored on a 5-point Likert-scale (from strongly agree to strongly disagree) and reflects the therapist’s impression of the practicability of the device. We calculated the SUS scores for each therapist and the average SUS score over all therapists. We transferred the average SUS score also to a percentile score, where a value of 68 or higher is considered practical. Therapists further responded to the general question ‘How did you find Andago in general?’ and were asked to provide specific comments on their experiences with the Andago.

### Appropriateness

We investigated appropriateness in session 3 (Fig. 2). As we investigated differences in stride-to-stride variability of the stride time and lower limb inter-joint coordination between the Andago and treadmill trials, we needed to account for the influence of walking speed [24]. As it is not possible to walk in Andago at a pre-defined speed, we determined the self-selected walking speed during the Andago condition first and set the treadmill speed in the subsequent trial accordingly. This excluded the possibility to randomize the order of the conditions. After that, participants performed the other Andago trials with different levels of BWU in a randomized order. To investigate whether fatigue might have affected the results, participants performed the 10MWT at maximal speed (i.e., the instruction included now ‘… as fast as possible’) twice at onset and twice at the end of this session.

#### Technical equipment

Participants walked on the treadmill of our Lokomat® Pro V6 system. The BWU system shares similar characteristics as the Andago weight support system. Prior to the trials, patients were equipped with DTS 3D accelerometers (Noraxon U.S.A. Inc., Scottsdale/USA), self-adhesive hydrogel electrodes (Covidien, Mansfield/USA) to record sEMG, and DTS 2D flexible electrical goniometers (Biometrics Ltd, Newport, UK). Signals were transmitted with a sampling rate of 1500 Hz from the sensors to the MyoResearchXp software (Noraxon U.S.A. Inc., Scottsdale/USA) by the Wireless TELEmyo DTS (Noraxon U.S.A. Inc., Scottsdale/USA). Accelerometers were mounted on the patient’s left and right shoe over the Achilles-tendon region to detect initial contact and toe-off, which was needed to determine gait phases and cycles. EMG electrodes were positioned in line with the SENIAM guidelines [25] on the M. Gluteus Medius, M. Biceps Femoris, M. Gastrocnemius Medialis, M. Vastus Medialis, and M. Tibialis Anterior of the more affected leg. Still, we present only data of the antigravity muscles. The location was marked with a skin pencil, the skin was shaved, lightly rubbed with an abrasive gel to decrease skin impedance, and the electrodes were fixed. The participants were asked to contract each muscle to test the quality of the signal [25]. The three goniometers were mounted so that the middle of the goniometer was placed over the center of rotation of the hip, knee, and ankle joint of the more affected leg and calibrated.

We further used a Logitech HD Webcam C270 (Logitech Europe S.A., Nijmegen, Netherlands) to record the participants’ feet from behind during walking. The signal was transmitted with a sampling rate of 30 Hz via USB cable to the MyoResearchXp software. These synchronized video recordings of the feet enabled us to determine initial contact and toe-off for each Andago and treadmill trial in case of unclear accelerometer signals.

#### Differences between Andago and treadmill walking

The participants performed all measurements wearing their regular orthoses. The Andago trials were performed in the straight-line mode. We first measured the walking pattern in the Andago at the reference level of BWU (BWUref), i.e., the minimal amount of BWU needed for the participant to keep a physiological knee position (or as physiological as possible) during stance. This is also the BWU level used during therapeutic training sessions. We determined BWUref as follows. Participants were secured in the Andago harness and stood on the digital scale. Weight was unloaded until they could hold a physiological knee position.

Participants walked twice along a 17 m-long walkway at their self-selected pace. The time needed to cover ten meters was manually stopped with a stopwatch to calculate the mean speed. Between the trials, participants had one minute of rest. We asked the participants to walk without holding the handrails if this was physically possible.

Then, participants performed the treadmill trial (also at BWUref). We increased the treadmill speed until we reached the mean walking speed of the previous Andago BWUref trial. After six steps, we recorded 15 strides for data analysis. The treadmill was stopped, the participant had one minute of rest, and then the procedure was repeated.

#### Differences between walking at various bodyweight unloading levels

To investigate the influence of different levels of unloading, participants walked in the Andago at BWUref plus 15% additional BWU (BWUref + 15%) and BWUref plus 30% additional BWU (BWUref + 30%). The order of these conditions was randomized (www.randomizer.org/). The procedure was otherwise similar to the first Andago BWUref trial.

#### Data analysis

For each condition, we analyzed 30 ‘steady-state’ strides for each participant. A stride was defined as the time between two consecutive initial contacts of the measured leg and identified using the accelerometer signals and video recordings. We always analyzed an equal number of strides, as, for example, the stride-to-stride variability decreases with increasing numbers of strides [26]. Although a previous study in healthy adults recommended that 50 gait cycles are needed to evaluate the variability of spatiotemporal gait parameters reliably [26], we considered this too much for most of our young patients, and we agreed on 30 gait cycles, as this might be sufficient to achieve a more or less normal data distribution. Data were further processed in MATLAB R2016A.

#### Andago vs. treadmill walking

*Stride-to-stride variability:* We quantified the stride-to-stride variability by calculating the coefficients of variation (CV [%] = 100% x SD/mean) over the 30 stride times for each participant per condition.

*Inter-joint coordination:* To quantify the variability in inter-joint coordination, we calculated a normalized inter-joint coordination coefficient. For the joint signals, each of the 30 strides was normalized to 1500 data points. In line with Chiu et al., [27] the angular positions (x) were then normalized between 1 and −1 with the following formula to minimize differences in movement amplitude between conditions:

$${x}_{i, norm}= \frac{2 \times ({x}_{i}-\mathrm{min}({x}_{i}))}{\mathrm{max}\left({x}_{i}\right)-\mathrm{min}({x}_{i})}-1$$

Then, we averaged the normalized kinematics over the 30 strides to achieve an averaged stride for each joint. To obtain the inter-joint coordination variability, the magnitudes of the vectors pointing from the average stride to each of the other 30 strides on the knee-hip and knee-ankle angular position plots were calculated for each data point. The variability is represented by the mean of the magnitude of all vectors [normalized root mean square (RMS)].

*Different BWU levels in Andago:* For the EMG signals, we determined the stance-phase (time from initial contact to consecutive toe-off of the measured leg) for each of the 30 strides. Each stance phase was normalized to 900 data points. EMG data were rectified, filtered with a 20 Hz Butterworth high-pass filter [28], and smoothened by RMS with a time window of 50 ms. We calculated the mean EMG amplitude for each stride [μV] and then calculated the average amplitude over the 30 stance phases for each participant per condition.

#### Possible confounding of holding parallel bars

As some patients required the use of the parallel bars of the Andago or the treadmill, and different levels of support could influence specific parameters, we recorded the amount of pressure applied by the patients on the parallel bars. We mounted per handrail three 40 cm long (Andago) or 30 cm long (treadmill) FSR 408 pressure sensor stripes (Interlink Electronics, Westlake Village, USA). To ensure that the force applied by the patients was distributed entirely over the measurement area, we fixated long stripes over the active force-detecting part of the sensor stripes (Fig. 1b and c). We covered this with tape and inserted a tube of slightly larger diameter around the bar to focus the force distribution on the measurement area and to decouple the vertical support force (we were interested in) from circular grasping forces. The signal was transmitted via cable from the sensors to an Arduino MEGA 2560 microcontroller board and then to MATLAB R2016A (MathWorks Inc., Massachusetts/USA) with a frequency of approximately 5 Hz. Children who were not able to walk hands-free in a condition (also based on the previous two familiarization sessions) were asked to hold the handrails during all Andago and treadmill trials to ensure comparability between the conditions. We calculated the mean values of the forces produced with the right and left hand on the handrails over the two trials for each condition with MATLAB. We summed these values of the left and right hand and presented them as a percentage of the participant’s bodyweight.

### Statistical analyses

Statistical analyses were performed with the software R (Version 3.4.3, R Core Team, Vienna, Austria, 2017) and SPSS (Version 24, IBM).

We tested the distribution of the data with the Shapiro–Wilk test and Q–Q plots. Based on their outcomes, we performed merely non-parametric statistics. Differences between two dependent conditions were tested with two-sided Wilcoxon signed-rank tests (i.e., Practicability: the number of bumps when passing a door between session 1 and 2; Acceptability: differences between the first and second session; Appropriateness: stride-to-stride variability of stride time and inter-joint coordination). Data reflecting the accuracy of the BWU system compared to the digital scale (for 25%, 50%, and 75% of BWU) were normally distributed, so we performed paired t-tests. Alpha was set at 0.05.

Differences between the three Andago BWU conditions (Appropriateness) were tested with a Friedman’s test followed by pairwise Wilcoxon signed-rank tests. Here, we applied a Bonferroni’s correction for the pairwise comparisons and set alpha at 0.017.

## Results

### Participants

Twenty participants were recruited, but data were obtained from 18 children (ID17 walked too fast to walk in the Andago, and ID04 was not compliant enough and stopped after the first session). The 18 participants (7 girls, 11 boys) were 13.9 ± 3.0 years old (mean ± SD; range from 7.7 to 17.9 years), weighted 48.5 ± 14.2 kg (range: 21.5 to 84.2 kg), and were 1.56 ± 0.15 m tall (range: 1.22 to 1.85 m). In 11 children, the left leg was more affected, and in 6 children, the right leg. In 2 children who were bilaterally equally affected, we measured the left leg. Diagnoses, severity, and functional measures are presented in Table 1.

**Table 1:** Clinical and functional characteristics of the participants

ID	Diagnosis	Orthoses	Walking aids	GMFCS	MMT [x/80]	SCALE [x/20]	10MWT [s]	FMS 5 [x/6]	FMS 50 [x/6]	FMS 500 [x/6]	GFAQ [x/10]	WeeFIM mob	WeeFIM cog.
01	Bilateral spastic CP	–	Posterior walker	III	33	3	20.3	1	1	1	3	21	32
02	Brain tumor*	–	–	I	74	20	10.4	5	4	4	8	23	31
03	Critical illness polyneuropathy	Foot lifter bilateral	–	II	58	8	12.8	5	1	1	6	25	28
05	Bilateral spastic CP	–	–	II	59	7	9.9	5	5	5	8	33	31
06	Polytrauma after traffic accident	Static AFO left	Forearm crutches	II	NT	NT	13.7	5	3	3	8	25	30
07	Bilateral spastic CP	–	–	II	62	12	13.2	5	4	4	9	33	34
08	Guillan–Barré	Foot lifter bilateral	–	II	42	10	12.5	5	4	4	6	29	25
09	Traumatic brain injury	–	Posterior walker	III	62	15	58.0	2	2	1	4	20	20
10	Bilateral spastic CP	Static AFO bilateral	–	II	48	7	9.5	5	4	2	7	32	28
11	Cerebral ataxia	Insoles	–	II	56	11	39.4	5	4	1	5	24	29
12	Bilateral spastic CP	–	–	II	51	7	13.1	5	5	4	7	33	28
13	Cerebral ataxia	Dynamic AFO blateral	Posterior walker	IV	27	2	15.2	2	2	1	5	21	11
14	Bilateral spastic CP	Static AFO blateral	Anterior walker	III	39	6	16.5	2	2	1	6	29	35
15	Unilateral spastic CP plus Ependymom	Static AFO left	–	II	62	11	9.2	6	6	5	8	33	34
16	Cerebritis	–	–	II	NT	NT	9.2	5	5	5	8	33	25
18	Osteosarcoma	–	–	III	62	17	14.0	5	4	1	4	29	31
19	Bilateral spastic CP	–	–	II	48	9	11.3	5	4	2	7	16	26
20	Charcot–Marie Tooth syndrom	Dynamic AFO blateral	–	II	59	7	10.8	5	5	3	7	14	26
	Median (IQR)			2.0 (2.0–3.0)	57.0 (43.5–62.0)	8.5 7.0–11.8)	13.0 (10.3–15.5)	5.0 (4.3–5.0)	4.0 (2.0–5.0)	2.5 (1.0–4.0)	7.0 (5.0–8.0)	27.0 (21.0–33.0)	28.5 (25.8–31.3)

ID4 and ID17 were drop outs. *ID2 was blind. In two patients, MMT and SCALE sum scores could not be calculated because it was not possible to test single joints (in ID06 due to a skin lesion, in ID16 due to pain). Abbreviations: GMFCS, Gross Motor Function Classification System; MMT, Manual Muscle Test; SCALE, Selective Control Assessment of the Lower Extremity; 10MWT, 10 m Walk Test performed at preferred speed; FMS, Functional Mobility Scale; GFAQ, Gillette Functional Assessment Questionnaire Walking Scale; WeeFIM, Functional Independence Measure for children; mob., mobility; cog., cognition; CP, Cerebral Palsy;; AFO, anke–foot orthosis; NT, not testable; IQR, interquartile range.

### Practicability

**Fig. 3:**
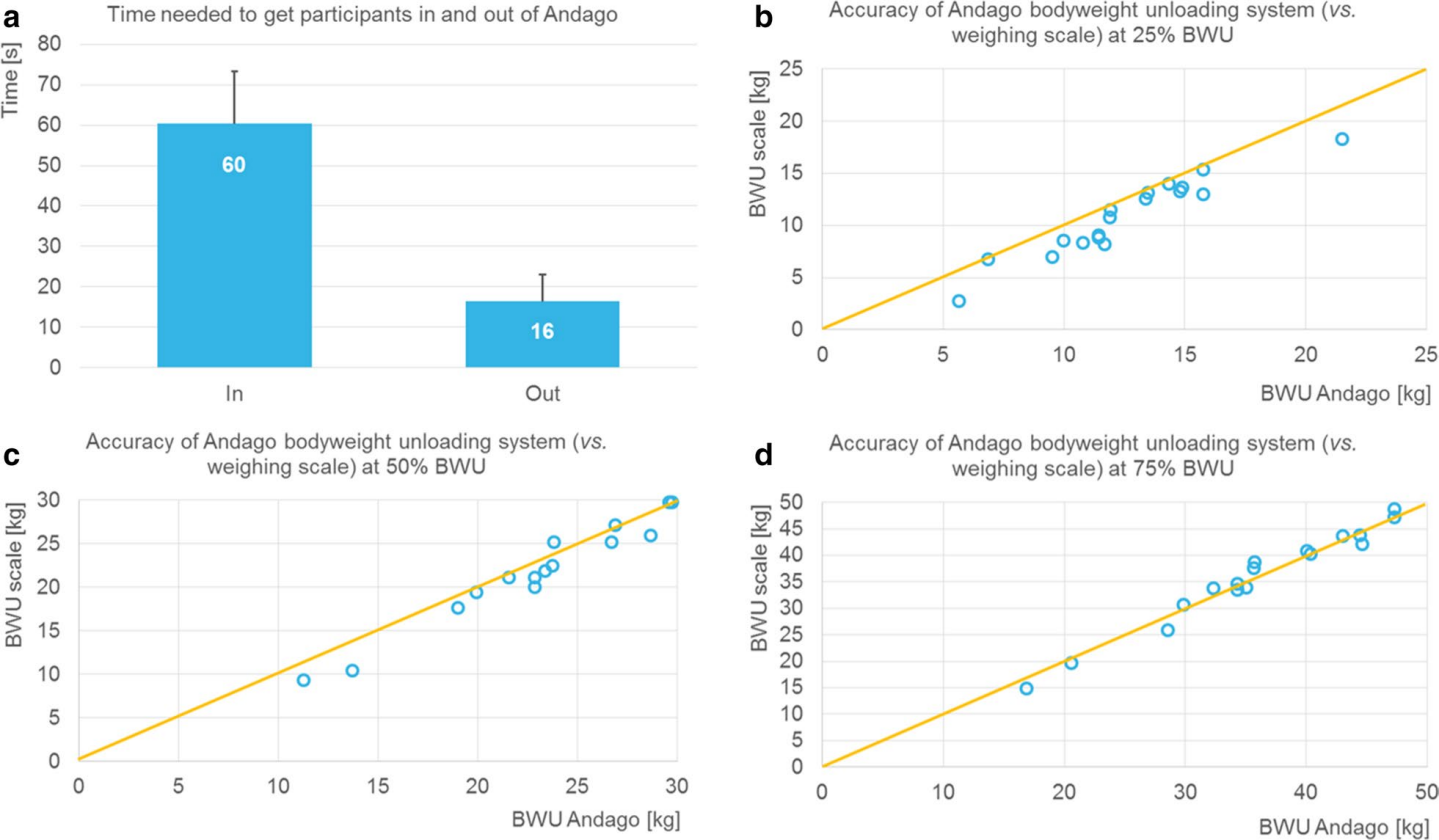
Practicability aspects of the Andago. **a** Average time [s] with standard deviation required to get patients in and out of Andago. **b**–**d** Comparison of the bodyweight unloading (BWU) as set in Andago compared to the actual values for conditions of 25%, 50%, and 75% of BWU, respectively. BWU, bodyweight unloading

Experienced therapists needed between 18 and 106 s to get participants into Andago and between 4 and 46 s to get them out. The average values are shown in Fig. 3a.

The results of the accuracy of the weight support of Andago compared to the digital weighing scale are shown in Fig. 3b to d (for the conditions of 25%, 50%, and 75% of BWU, respectively). The bodyweight of the children and the harness varied between 22.5 and 86 kg (mean ± SD is 50.0 ± 14.3 kg). We deleted one measurement for the 75% condition due to an error. All other measurements were complete. Data were normally distributed. For the 25% and 50% BWU conditions, the weighing scale reported significantly lower BWU compared to the Andago settings (25%: Andago 12.5 ± 3.6 kg, Scale 10.9 ± 3.7 kg, p < 0.001; 50%: Andago 25.0 ± 7.2 kg, Scale 23.7 ± 7.2 kg, p = 0.001). For the 75% condition, the results were similar between the Andago and the weighing scale. (75%: Andago 35.9 ± 8.7 kg, Scale 36.0 ± 9.2 kg, p = 0.88). The average relative deviations were −13.0% (25%BWU), −5.0% (50%BWU), and + 0.2% (75%BWU).

Concerning the other practicability aspects, we did not experience a device failure during the study. Andago prevented 12 times a fall (in 7 participants). Furthermore, Andago stopped for safety reasons 43 times in 11 patients (number of stops varied between 0 and 9 times per session and between 0 and 13 times per participant). Finally, bumps when passing through the door were not assessed in one patient due to blindness (ID02). The other 17 patients bumped between 0 and 5 times (of 5 times) against the door (session 1: median (IQR) = 5 (1–5); session 2: 4 (0–5); p = 0.047).

### Acceptability

**Fig. 4:**
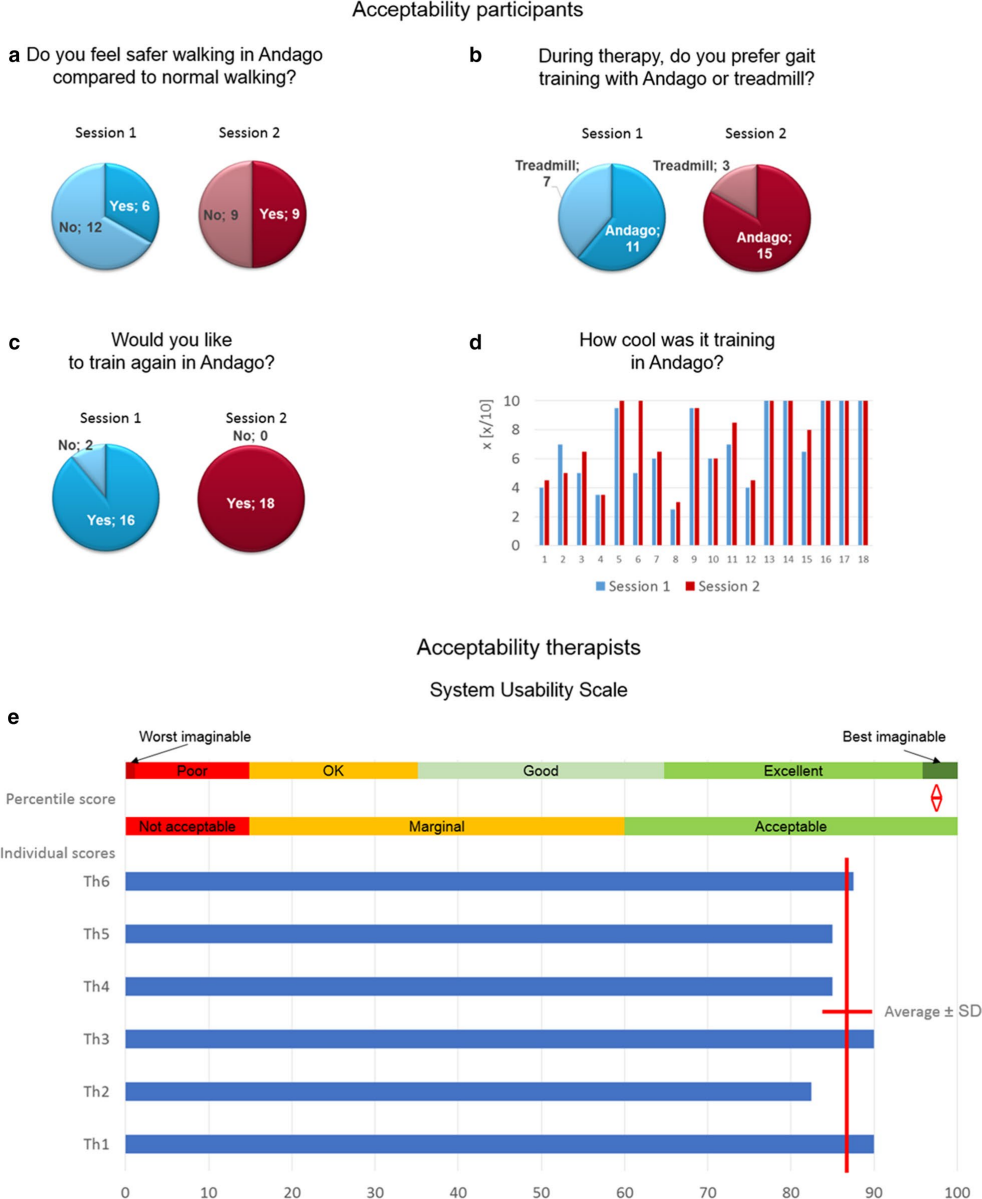
Acceptability of Andago by participants and therapists. Pie-charts showing the number of participants who **a** felt safer walking in Andago compared to normal walking, **b** preferred to train with Andago compared to treadmill walking, and **c** would like to train again with Andago. **d** Individual patient responses, whether it was cool training in Andago. Results from session 1 are colored in blue, from session 2 in red. **e** Individual System Usability Scale (SUS) scores for six therapists (Th1-6). Average and standard deviation (SD) are visualized with red crossing lines; the percentile score (displayed by the red up/down arrow) was calculated and interpreted in line with Bangor et al. [29]. *SD* standard deviation, *Th* therapist

Acceptability of Andago improved in the participants from the first to the second session (Fig. 4a–c). The median (IQR) level of coolness to train in Andago tended to improve from 6.75 (4.75–10.00) in session 1 to 8.25 (4.88–10.00) in session 2 (p = 0.055, Fig. 4d).

Six therapists (5 females, 1 male; 3 health/movement scientists, 3 physiotherapists) filled out the SUS questionnaire. Their experience in working therapeutically with children varied from 1.5 to 14 years (median 3 years and 3 months). Their experience in applying rehabilitation technologies to children varied between 5 months and 7 years (median 2 years and 3 months). The SUS percentile scores of the therapists were high, with an average of 86.7 ± 3.0 (depicted with the red crossing lines in Fig. 4e), which lead to a percentile between 96 and 100%, which is considered ‘acceptable’ or ‘best imaginable’ [29]. Two therapists responded that they liked the Andago very much, and four that they liked it. Individual responses were:

- ‘Uncertainties in use are more related to the environment and not to the device. If the space is very narrow, the sensors do not react quickly enough (or miss something), and there is a risk of collision with objects, walls, doors, etc.’
- ‘With Andago, you can perform various exercises of ‘conventional physiotherapy’ without the risk that a patient would fall. Children may dare to try out new movements more when they are secured in Andago than during a regular physiotherapy session.’
- ‘Depending on the diagnosis and objective, the use of the Andago in a therapeutic environment is very beneficial. I had the impression that especially children who do not take much weight on their legs in everyday life, or who had specific goals, liked to train with the Andago. From a therapeutic point of view, it was very interesting to observe the gait pattern and, e.g., changes caused by interventions (wedge, orthosis…).’
- ‘Depending on the room, it is more or less difficult to maneuver safely with Andago, especially in the patient following mode, where children have more spatial freedom.’
- ‘In my opinion, the therapeutic Andago sessions and the conventional physiotherapy sessions should be better ‘synchronized’ to optimize the therapy of the child, for example, to enable a high number of repetitions of specific movements in both Andago and conventional therapy.’
- ‘Regular use of the device is important for user safety. I sometimes had no Andago-training for a long time, and when I had a training session, I needed some time to know exactly how the device worked.’
- ‘Patients reported that they could train closer to reality in Andago compared to training on a treadmill. They considered training in Andago as qualitative higher and requiring more concentration compared to on a treadmill.’

### Appropriateness

Half of the patients (n = 9) needed to hold on to the parallel bars. The time needed to perform the 10MWT at maximum speed at onset and end of session 3 was not significantly different (median (IQR) at onset: 8.9 s (7.4–13.5 s); afterward: 8.7 s (7.2–12.1 s); Wilcoxon signed-rank test: p = 0.07).

#### Andago vs. treadmill walking

**Fig. 5:**
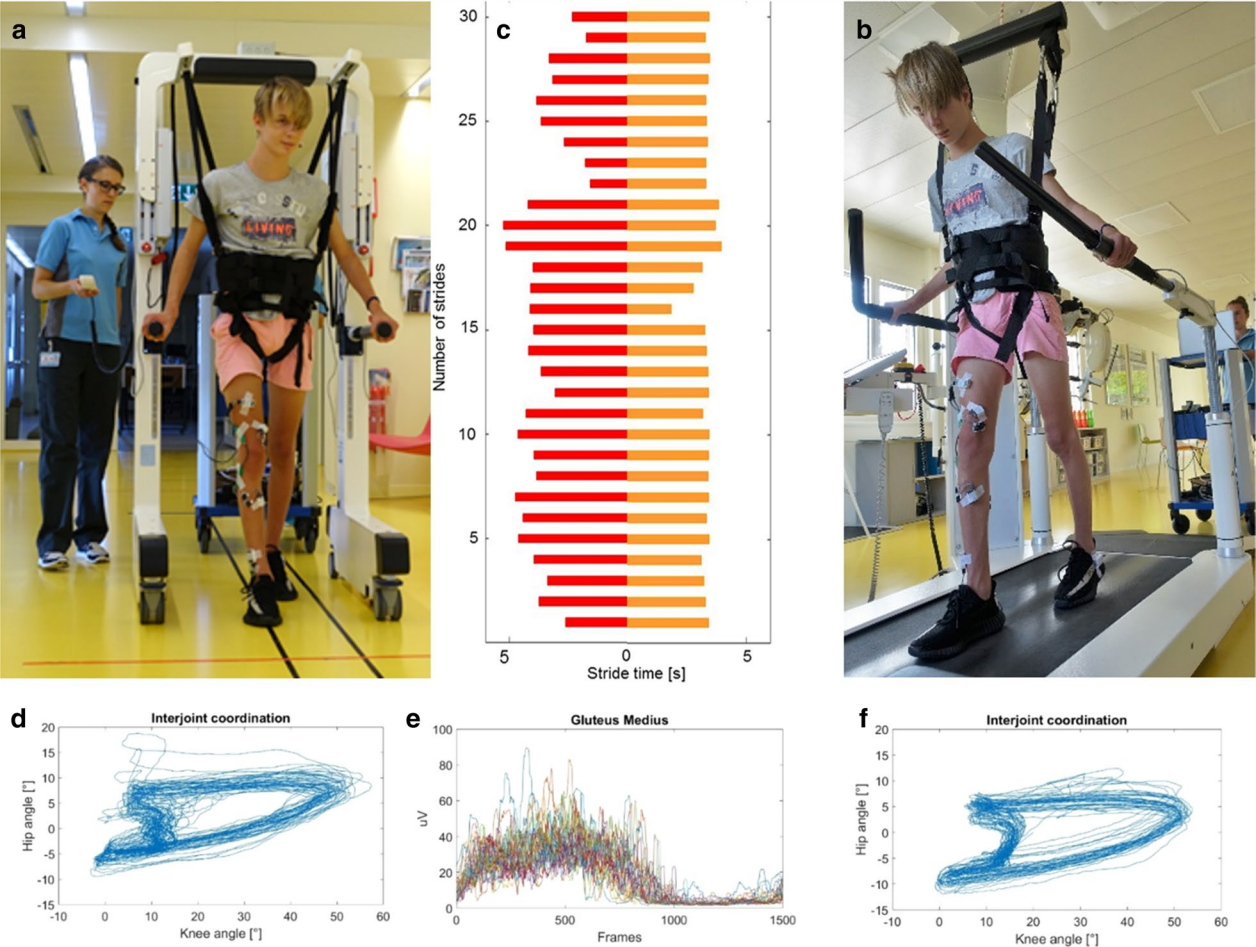
Individual example. Adolescent participant walking **a** in the Andago and **b** on the treadmill. Stride-to-stride variability of the stride time between **a** and **b** is shown in (**c**; red for the Andago, orange for the treadmill). Variability over 30 strides in hip-knee inter-joint coordination (**d** and **f**) and (**e**) muscle activity of the M. Gluteus Medius. The participant and his parents provided (written) consent for publication

Figure 5 shows an example of a patient walking (a) in Andago and (b) on the treadmill. Also shown are (c) the 30 stride durations, (d and f) cyclograms reflecting the hip-knee inter-joint coordination, and (e) muscle activity patterns and amplitudes of the M. Gluteus Medius (only for Andago condition).

**Fig. 6:**
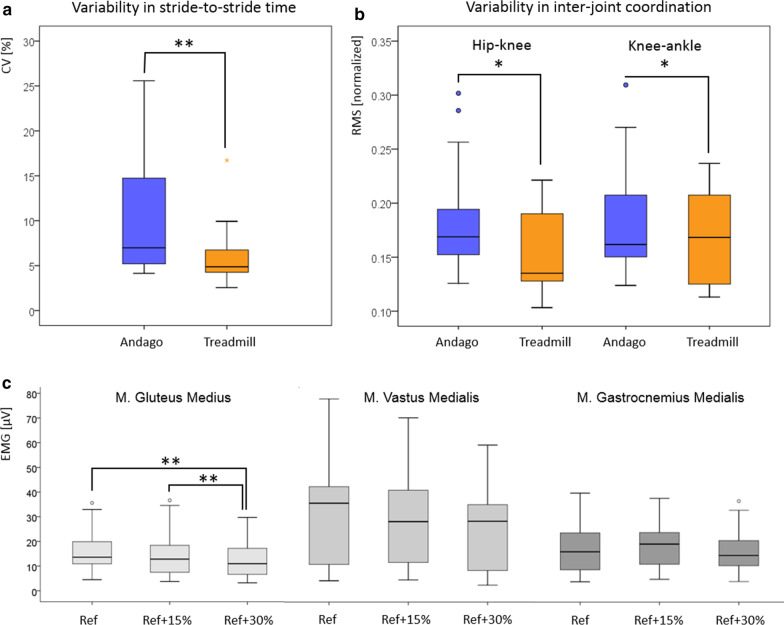
Appropriateness of Andago. **a** Participants walked with higher stride-to-stride variability in stride time in Andago when compared to on the treadmill. **b** Also, the variability in inter-joint coordination between the hip and knee and the knee and ankle were higher when walking in Andago compared to the treadmill. **c** Antigravity leg muscle activity during the stance phase. *CV* coefficient of variation, *RMS* root mean square, *EMG* electromyography, *M.* Musculus, *Ref* reference bodyweight unloading (BWU); Ref + 15% or Ref + 30%, reference BWU plus additional 15% or 30%, respectively

While averaged stride times were comparable between the Andago (median 1.5 s, IQR 1.1–1.8 s) and the treadmill (median 1.5 s, IQR 1.3–1.8 s, p = 0.59), the CVs reflecting the stride-to-stride variability were significantly higher in Andago (median 7.0, IQR 5.1–15.1) compared to the treadmill condition (median 4.9, IQR 4.1–6.9, p = 0.002, see Fig. 6a).

The variability of the inter-joint coordination was quantified by RMS values. The hip-knee inter-joint variability (Fig. 6b) was higher in the Andago condition (median 0.17, IQR 0.15–0.21) compared to the treadmill condition (median 0.14, IQR 0.13–0.19, p = 0.011). Despite comparable median knee-ankle inter-joint variability values, variability was also higher in the Andago (median 0.16, IQR 0.15–0.22) compared to the treadmill (median 0.17, IQR 0.12–0.21, p = 0.039, Fig. 6b).

*Different BWU levels in Andago:* We could obtain EMG measurements of the M. Gluteus Medialis in 18 participants, of the M. Vastus Medialis in 17 (one loss due to poor EMG quality), and of the M. Gastrocnemius Medialis in 13 participants (5 participants wore lower leg orthoses, which prohibited fixation of the electrodes at the correct position). Friedman tests showed a significant difference between the three BWU conditions for the M. Gluteus Medius (Chi-Square = 14.8, p = 0.001), but not for the M. Vastus Medialis (Chi-Square = 3.3, p = 0.19) and M. Gastrocnemius Medialis (Chi-Square = 1.4, p = 0.50, see Fig. 6c). EMG activity of the M. Gluteus Medius was significantly lower in the BWUref + 30% condition compared to the BWUref (p = 0.002) and BWUref + 15% (p = 0.003) condition. The difference between BWUref and BWUref + 15% showed a trend towards a lower activity when unloading with an additional 15% (p = 0.022).

#### Possible confounding of holding parallel bars

The median force that the nine participants exerted on the parallel bars amounted to 6% of their body weight (IQR 4.5–15.3%) in Andago (BWUref) and 3.3% (IQR 2.5–10.5%) on the treadmill. The difference was not significant (Wilcoxon signed-rank test: p = 0.09). The median forces during the other BWU conditions in Andago were well comparable and not statistically different from the BWUref condition (BWUref + 15%: 6.5%, IQR 4.8–14.7%; BWUref + 30%: 5.9%, IQR 4.4–11.3%; Friedman’s test: p = 0.72).

## Discussion

In this clinical utility study, we investigated the practicability, acceptability, and appropriateness of the over-ground BWU robotic device Andago in children and youths with neuro-orthopedic disorders. While we are aware of some studies that evaluated walking with a motorized over-ground BWU device in adult patients after stroke [30, 31], to our knowledge, this is the first study providing a comprehensive evaluation of the clinical utility of such a technology in a heterogeneous group of young patients reflecting the patient population that our therapists treat daily.

### Practicability

Several findings support the practicability of the Andago device. The short time needed to get patients in and out of the device is a relevant factor when deciding whether such a technology can be integrated into a rehabilitation center. While this time might be initially higher for therapists without prior experience in applying the Andago, based on our clinical experience, this familiarization occurs relatively rapidly, particularly if therapists are experienced in performing transfers. The large differences between patients merely depended on the ability to stand independently. For patients who required support and were positioned out of or back into a wheelchair, more time was needed. Indeed, when correlating the average time needed to get participants in the Andago with gait performance measures like the GFAQ or FMS, we found negative Spearman correlation coefficients of moderate magnitude with a tendency to statistical significance (GFAQ: ρ = −0.46, p = 0.054; FMS over 500 m: ρ = −0.44, p = 0.066). Correlations might have been higher if we had included an assessment measuring standing ability. As the device also does not require much time to start or shut down and a relatively small number of control modes allowing individual adjustments are available, we consider time not a limiting factor for applying the Andago to patients in a specific robotic-therapy session or even using it spontaneously during a physiotherapy session.

The BWU system seems to function well, especially at higher levels of unloading. This is particularly important for patients after orthopedic surgery who might at a certain time post-surgery increase load on the operated leg or foot. At lower levels of unloading, deviations were larger, which can be explained by the weight and friction of the system. While the company reported relative deviations of + 3% (at 10 kg unloading) and + 14% (at 25 kg), we found small yet negative relative deviations at 25% and 50% BWU. Negative deviations indicate that the unloading in Andago was significantly smaller compared to our comparator measurement. We need to consider that the system includes a relative crude BWU scaling, making it difficult to set low levels of BWU (children participated, weighing as few as 21.5 kg) accurately. Overall, the accuracy is sufficient for clinical applications where the unloading will be set by the patient’s performance and not by numbers. Especially for those patients who are in fear of loading their leg or in fear of pain, the Andago is a very useful tool.

Concerning the other practicability aspects, the Andago performed reliably during the duration of the study, and we did not encounter a technical failure. During the study, Andago prevented 12 times that a patient would fall and automatically stopped 43 times if patients had moved too fast. We consider this relevant because of two reasons. First, the Andago allows a safe therapy because, without the device, only a therapist might have prevented these falls. Second, patients seem to train at the limit of their abilities. Indeed, rehabilitation therapy is all about pushing the sensorimotor system step by step a bit further. Psychological studies have shown that patients with developmental delays seem less motivated and show a more passive playing behavior (less complex and challenging) than peers despite equal curiosity and pleasure [32, 33]. We assume that in some patients, the Andago provided the feeling of safety, which is partly in line with our patients’ acceptability results and might have motivated them to move closer to their limits of stability.

### Acceptability

A qualitative study investigating the expectations and experiences of children with CP about robotic gait training using the Lokomat Pro showed that these children did not consistently feel excited about, have a wish to use, or have a sustained interest in the use of robotic technologies, and at times experienced some anxiety about their participation in the intervention [34]. Also in our study, there was initial skepticism in some children in using the technology. Initially, most children felt safer when walking with their walking aids instead of walking in Andago. Some children reported that when they repeatedly bumped into the door frame, they got a bit ‘afraid’ of walking in the device. We had the impression that this affected the feeling of safety of the children. This underlines that it is important to initially prepare children for walking with such rehabilitation robots and introduce such technology, particularly during the first training sessions, to each child individually. Acceptability improved from session 1 to 2, showing that getting familiarized with training in the device improved the acceptance, and finally, all participants expressed the desire to train further with the device.

The scorings of the SUS reflecting the practicability and acceptability of the device by our therapists were high. Indeed, generally, the Andago is relatively easy to use and does not require many individual settings, as compared to, for example, a Lokomat. Therapists who have some experience in applying rehabilitation technologies will not require lengthy training to apply the device to a patient. Nevertheless, one therapist mentioned that a certain amount of time is needed to get used to the device again after it has not been used for a long time.

### Appropriateness

We could confirm our first hypothesis that participants walked with an increased stride-to-stride variability of the stride duration during over-ground walking in Andago compared to treadmill walking. Although the more considerable amount of variability in temporal gait parameters during over-ground walking was expected according to the previous literature in healthy adults [9] or adult patients with a hemiparetic stroke [10], it is interesting to find such a large effect also in our small and heterogeneous sample of children with gait impairments. The discrepancies between the two walking conditions are considerable. Not only the stride-to-stride variability during treadmill walking within a patient was reduced, but also differences between the participants were minimized. As gait speed is related to stride duration [35], the results can be explained by the variation in walking speed that is only possible in Andago and not on the regular treadmill, where the belt defines the pace, limiting the modulation of temporal gait parameters.

In a previous publication [36], the mean CV of step duration during self-selected walking amounted in 15 children with CP to 10.7%, in 15 children with traumatic brain injury to 13.0%, and in 30 typically developing children to 7.7%. In our patients, the average CV was 10.5% (median 7.0%) when walking in Andago and 5.9% (median 4.9%) when walking on the treadmill. Numbers between our patients walking in Andago and the children with CP and traumatic brain injury are comparable, but walking on a treadmill seems to reduce the stride-to-stride variability even below values obtained in typically developing children walking over-ground.

The variability in inter-joint coordination was higher for walking with the Andago compared to treadmill walking, especially in the hip-knee inter-joint coordination. Our finding of lower variability during treadmill walking is supported by the results of Dingwell et al. [37] or Chiu et al. [38], who found lower inter-joint coordination variability during the stance phase (but not during the swing phase) during treadmill walking. They concluded that the treadmill imposes a systemic regulation on dynamic neuromuscular control during walking. While Chiu and Chou [27] reported that the walking speed influenced the inter-joint coordination variability in healthy young adults, this should not be the source of the variability in our study, as the walking velocity was kept similar between the two conditions.

When comparing different levels of BWU, we could only partly confirm our hypothesis because only the activity of the M. Gluteus Medius was significantly reduced during high levels of BWU. While the dorsal part of the M. Gluteus Medium has an extensor and an antigravity function, its primary function as a hip abductor is ensuring pelvic stability preventing the pelvis from dropping on the swing leg’s side during walking. Mun et al. [39] found in healthy adults walking over-ground with a novel BWU robot with pelvic motion support a significant reduction in M. Gluteus Medius activity for 30% or 40% BWU (compared to 0%). The authors assumed that increased levels of BWU decrease the forces on the pelvis at initial contact and increase stability over the stance-phase. This can also apply to our participants, who required less M. Gluteus Medius activation to stabilize their gait. In our study, the activity of the M. Vastus Medialis and M. Gastrocnemius Medialis did not reduce significantly with increasing levels of BWU (Fig. 6c). Also Mun et al. [39] found no change in the M. Vastus Medialis, but they reported a significant reduction of M. Gastrocnemius Medialis activity at 20%, 30%, and 40%. A reduction was also found by Awai et al. [11]. They reported reduced M. Gastrocnemius Medialis activity at 50% BWU (not 30%) during the stance phase in healthy young adults walking in the FLOAT. Fenuta and Hicks [40] found in healthy non-disabled adults who walked in the Zero G system at different levels of BWU over-ground that offloading significantly decreased activity of the M. Gastrocnemius Medialis at 40% BWU and higher. Fischer et al. [12] found reductions in peak activity (during the stance-phase) of the M. Gastrocnemius Lateralis and M. Vastus Lateralis when comparing 15% with 0% BWU. We assume that the lower bodyweight of our participants could have partially caused the lack of a statistically significant reduction in muscle activity during the stance-phase of the M. Vastus Medialis and M. Gastrocnemius Medialis. A relatively small absolute change in unloading might have affected the muscle activity level to a lesser degree (compared to the findings obtained in adult participants). Especially for the M. Gastrocnemius Medialis, limited statistical power in our study might be an additional reason, as we could not obtain sEMG data of all participants. Several children wore orthoses, which made it not possible to measure this muscle. It is unlikely that differences between conditions can be explained by different levels of support exerted by the participants on the parallel bars, as the amount of pressure did not differ between the BWU conditions.

### Clinical experiences and relevance

Generally, our clinical experience with the device confirms most of the practicability and acceptability results of this study. Unlike any passive support system, Andago provides dynamic bodyweight unloading (i.e., the bodyweight unloading remains similar throughout the gait cycle despite the upwards and downwards movements of the patient). The bodyweight support can also be adjusted during walking. Furthermore, the device follows the patient relatively transparent, and the therapist does not need to push the device and can focus entirely on the patient. Concerning safety, we never experienced a critical incident during the past four years. In single conditions, the therapist who is always accompanying a child during Andago therapy could prevent a potentially unsafe situation using the remote controller or safety button. We rarely had any technical issues during the past four years, except for the battery, which was recently replaced.

While the practicability and acceptability results are overall very positive, we also experienced limitations of Andago, especially when preparing this clinical utility study. Unobstructed walking and turning in Andago requires space, so we had to select a location in our rehab center that was spacious enough. Some of the therapists mentioned this also in their notes complementing the SUS questionnaire. Space requirement was also reflected in the difficulties patients experienced when stepping through the door. Despite that doors in our rehab center are broader than regular doors, many patients bumped into it. Although there was a decrease in the average number of bumps in the second session, indicating familiarization with the Andago, the median of four bumps out of five trials in session two shows that it remained challenging to navigate in limited space with the device. We also had to take into account the floor surface. All measurements were performed on an even, hard, and smooth surface. Andago has difficulties following a person smoothly on uneven, irregular, or less firm surfaces like a carpet. According to the company, walking on ramps is not allowed, and doorsteps need to be less than 1 cm in height. Due to these spatial limitations, we consider it difficult to train gait endurance in our setting with Andago but include Andago in therapies focusing on various other walking-related goals.

Clinically, the appropriateness results are meaningful. First, they indicate that temporospatial gait characteristics during walking in Andago with BWU seem more similar to walking over-ground than treadmill walking with BWU. It can be discussed how much variability is desirable during training. On the one hand, earlier studies in the field of spinal cord injury aimed to stimulate central pattern generators (CPGs) by inducing very repetitive steps when walking on a treadmill (for a review, see [41]). On the other hand, studies in the field of motor learning promote repetition-without-repetition, i.e., a certain amount of variability is needed [42]. These should improve generalization or transfer of the learned task to daily life relevant conditions. While we have no definite answer to the question of how much variability is desirable during training with BWU, we assume that reducing variability in patients below levels observed for typically developing children cannot be beneficial for improving over-ground walking under daily life conditions.

We hardly train patients in Andago with levels of BWU exceeding 50% of the bodyweight (about BWUref + 30%), which makes us feel confident that most antigravity muscles are sufficiently trained. Reasons why we prefer to stay below 50% BWU are manifold: (i) higher levels of BWU induce painful sensations in the children when the harness is pulling them up, (ii) children walk more on their toes, which we need to avoid, as toe walking is part of the crouch gait pattern already, (iii) it makes it more difficult to train the push-off phase, (iv) the walking ability of most patients practicing in Andago is good enough to practice with less BWU. Patients who are more severely impaired often require guided leg movements and are therefore trained in a device like the Lokomat. Indeed, if the impairments of the patient allow it, we recommend staying below 50% BWU to ensure that also the important M. Gluteus Medius becomes sufficiently activated.

Motivation during training appeared for most participants high, but not for all. To keep motivation high also when repeatedly training, therapists include objects, which patients have to step on, over, or circumvent, to challenge them more. This should also improve the adaptability of the walking pattern to more challenging daily life environments.

Finally, one advantage of most new rehabilitation technologies is improved documentation of parameters reflecting training dosage. Andago can record the training duration, movement duration, covered distance, average training speed, and the number of prevented falls. The number of prevented falls was sometimes wrongly recorded when participants moved too fast (e.g., when they turned too quickly). As we hardly use the Andago device to improve gait endurance, we consider several of these parameters less relevant. However, in the long-term, a more detailed documentation of additional parameters reflecting training intensity (e.g., including the level of bodyweight support) or gait quality (e.g., the variability in stride length, symmetry of steps, or accuracy when following a predefined path in the patient-following mode), might be beneficial to monitor more precisely improvements in the walking ability of the patients.

### Methodological considerations

Some of the questions the participants had to answer during the acceptability part (for example, ‘How cool was it training in Andago?’) were not validated standardized questions. Yet, we decided to use such formulations because questions from validated standardized assessments are often too difficult for our patients. Another consideration is that we applied a classification system like the GMFCS or an assessment like the SCALE to all participants, despite that these have been psychometrically evaluated only for children with CP. We applied these to achieve comparable cross-sectional indicators for mobility (GMFCS) or lower limb selective motor control (SCALE), as similar measures for patients with other diagnoses are missing.

Many participants had a relatively good walking ability, despite some impairments in lower limb functions and limitations in walking. This selection of relatively good patients is partly caused by the Andago device itself (e.g., in contrast to a motorized exoskeleton device like a Lokomat, leg movements need to be initiated and performed by the patient), but should be considered when generalizing the results from this clinical utility study.

While therapists filled-out the SUS for the Andago, we did not assess the usability of another similar device (e.g., the RYSEN) systematically as a control. Such a comparison would have allowed us to put the usability of the Andago into the perspective of other technologies.

As walking speed can influence various temporospatial parameters and muscle activation levels, we used the same speed for the over-ground and the treadmill trials. This, although healthy adults walked at a slower self-selected speed on the treadmill compared to over-ground [38]. Therefore, our participants might have walked on the treadmill at slightly higher than preferred speeds, which could have affected some of the parameters and responses of the participants on the acceptability questions.

Due to limited space and limited walking endurance of some patients who could not perform 30 strides without a break, we collected the stride-to-stride variability over two separate walking trials. This, although it was shown that reliable information about gait variability should be collected during continuous walking [26]. As this was done over all conditions (Andago and treadmill), we think that it should not have affected the observed results to a large degree.

We adapted our BWU levels relative to the ability of each patient. While other studies always applied fixed percentages of BWU, we considered our approach more meaningful for patients, as BWUref can be considered equivalent to the ‘0% BWU’ of healthy participants. However, this might have influenced our results and the comparisons to other studies, as it induces a large variability in BWU within a condition. For example, the percentages of BWU varied between 6 and 42% of the bodyweight within the BWUref condition.

The clinical utility model, according to Smart [8], also includes accessibility, which is an important topic when deciding on acquiring new technology. While we did not investigate accessibility in this study, we can report that we purchased the Andago in 2016 for around CHF 60′000.-. Annual maintenance is not required, but if desired a maintenance contract can be made with the company. As the effectiveness of the Andago has not been shown, it is currently not possible to determine the cost-effectiveness. Concerning financial reimbursements for such interventions, these differ between countries and insurance companies. For example, the current situation in Switzerland is that we receive a lump sum per day for each patient undergoing inpatient rehabilitation, so we do not receive additional reimbursement for applying rehabilitation technologies.

## Conclusions

Based on the current results, we consider the Andago a practical and well-accepted device to train walking over-ground in patients with gait impairments. In contrast to walking on a treadmill, the system allows for walking with BWU without reducing individual stride-to-stride variability of temporospatial parameters. When walking at levels of BWU as investigated in our study, the levels of sEMG activity of several antigravity muscles was not largely affected.

## Data Availability

The datasets generated and/or analyzed during the current study are not publicly available.

## Abbreviations

10MWT: 10-M Walk test
AFO: Ankle–foot orthosis
BWU: Bodyweight unloading
BWUref: Reference level of bodyweight unloading
BWUref + 15%: BWUref plus 15% additional bodyweight unloading
BWUref + 30%: BWUref plus 30% additional bodyweight unloading
CP: Cerebral palsy
CPG: Central pattern generator
CV: Coefficient of variation
FMS: Functional Mobility Scale
GFAQ: Gillette Functional Assessment Questionnaire Walking Scale
GMFCS: Gross Motor Function Classification System
ICF-CY: International Classification of Functioning, Disability and Health—Children and Youth
IQR: Interquartile range
MMT: Manual Muscle Test
NT: Not testable
RMS: Root mean square
SCALE: Selective control assessment of the lower extremity
SD: Standard deviation
sEMG: Surface electromyography
SUS: System usability scale
Th: Therapist
WeeFIM: Functional Independence Measure for Children
